# Postoperative symptom changes following uterine artery embolization for uterine fibroid based on FIGO classification

**DOI:** 10.1186/s42155-025-00520-7

**Published:** 2025-01-29

**Authors:** Yoshimi Nozaki, Shiori Takeuchi, Masafumi Arai, Yoshiki Kuwatsuru, Hiroshi Toei, Shingo Okada, Hitomi Kato, Naoko Saito, Takamichi Nobushima, Keisuke Murakami, Mari Kitade, Ryohei Kuwatsuru

**Affiliations:** 1 Department of Radiology, Graduate School of Medicine, Juntendo University, 2-1-1 Hongo Bunkyo-Ku, Tokyo, 113-8421 Japan; 2 Department of Obstetrics and Gynecology, Graduate School of Medicine, Juntendo University, 2-1-1 Hongo Bunkyo-Ku, Tokyo, 113-8421 Japan

**Keywords:** Fibroid, Menstrual complications, FIGO staging, Uterine artery embolization, Treatment response

## Abstract

**Background:**

Classifying uterine fibroid using the International Federation of Gynecology and Obstetrics (FIGO) classification system assists treatment decision-making and planning. This study aimed to study whether different fibroid locations influence clinical outcomes following uterine artery embolization (UAE).

**Methods:**

This is a retrospective cohort study of patients who underwent UAE for symptomatic uterine fibroid between December 2016 and January 2023 at our hospital. Changes in mean fibroid volume were compared based on MR images. Menstrual pain, excessive flow symptoms, and treatment satisfaction before UAE and 6 months after UAE were compared.

**Results:**

A total of 149 premenopausal patients (mean age 45.7 ± 2.7 years) were included for analysis (FIGO 2/3, *n* = 57; FIGO 4–7, *n* = 92). Baseline menstrual pain, fibroid, and uterine volume before UAE were comparable between the two FIGO groups (*p* > 0.05). The menstrual flow index was higher for the FIGO 2/3 group (mean** ± **SD [min–max]: 9.4 ± 1.4 [4–10] *vs* 8.0 ± 2.3 [0–10], *p* < 0.001). Six months after UAE, the improvements in menstrual flow index (mean** ± **SD]: -3.7 ± 2.6 *vs* -2.6 ± 2.2, *p* = 0.035), fibroid volume (mean** ± **SD: -54.7 ± 21.7% *vs* -39.8 ± 16.2%, *p* < 0.001), and uterine volume (mean** ± **SD: -38.2 ± 16.3% *vs* -31.1 ± 11.6%, *p* = 0.008) in the FIGO 2/3 group were significantly higher than the FIGO 4–7 group. Both groups had comparable improvements in menstrual pain index (-2.1 ± 2.6 *vs* -1.8 ± 2.5, *p* = 0.008) and 88% of the patients were satisfied or very satisfied overall.

**Conclusion:**

UAE treatment satisfaction was high for patients with fibroids at different FIGO stages. UAE treatment outcomes were better for patients with fibroids affecting the endometrium (FIGO 2/3).

**Level of evidence:**

3B, Retrospective observational study.

**Supplementary Information:**

The online version contains supplementary material available at 10.1186/s42155-025-00520-7.

## Introduction

Uterine fibroids, also known as uterine myomas or leiomyomas, are benign tumors consisting mainly of smooth muscle cells. Although non-malignant, uterine fibroids can be debilitating for women, causing menstrual disorders, infertility, pregnancy complications, and bulk symptoms that negatively impact women’s quality of life [1]. A recent study conducted in the United States found that among 2,994 women suffering from uterine fibroid-related conditions, the healthcare burden amounted to 6,585 medical encounters and 625 hospital bed days in 2022 [2]. In Japan, a current history of uterine fibroid was an independent risk factor for incidental anemia found in women aged 35 years and older during annual health check-ups [3]. Treatment modalities for symptomatic uterine fibroids include medication, surgery, and minimally invasive procedures including uterine artery embolization (UAE) to control abnormal menstruation, reduce lesion size, or cure the disease definitively [4]. For women who prefer uterus-sparing treatments and are not seeking to preserve fertility, UAE is a viable treatment option that decreases both fibroid volume and symptom severity, and the feasibility of a same-day UAE protocol has been demonstrated to be safe and effective in the outpatient setting [5]. Studies that utilize the COMPARE-UF registry have found improvements in health-related quality of life (HR-QoL) and symptom severity scores, which persisted for 2–3 years after UAE and were comparable to myomectomy [6, 7]. Multicenter randomized trials such as the Embolization versus Surgical Treatment for Fibroids (REST-trial) have reported comparable improvement in HR-QoL and in levels of satisfaction at 1 year [8, 9], and the HR-QoL remained comparably stable 10 years after UAE or hysterectomy from the EMMY trial [10].

In 2011, the International Federation of Gynecology and Obstetrics (FIGO) introduced a fibroid classification system that categorizes fibroids based on the lesional involvement of submucosal, intramural, and serosal layers of the uterus [11]. FIGO classification for uterine fibroid is critical for making management plans clinically and is conducted based on imaging-derived (i.e., transabdominal, transvaginal ultrasonography, or magnetic resonance imaging [MRI]) datasets, including estimation of total uterine volume and number of fibroids [12]. Among the different types of uterine fibroids, those with submucosal and intramural components extending into the uterine cavity are considered to affect fertility the most [13]. Fibroids of FIGO types 0–2 may cause menorrhagia, dysmenorrhea, infertility, and pregnancy loss due to their protrusion into the endometrial cavity, and are treated with techniques such as hysteroscopic myomectomy and UAE to relieve symptoms [14, 15]. FIGO 3 fibroids have been associated with lower rates of live births, pregnancies, and implantation during fertility treatments, and a careful myomectomy without endometrium disruption is the preferred treatment approach in women who wish to preserve fertility [13]. Fibroids of other types are related to menstrual and bulk symptoms, and volume reduction or resection via UAE and laparoscopic/open myomectomy may be considered as these fibroids are difficult to reach with hysteroscopy [14, 15]. Reports of UAE efficacy across different types of fibroid have been published [16]. However, despite the utility of FIGO staging in selecting treatment, particularly UAE, there is limited evidence-based analysis of the role of FIGO classification in the real-world setting.

Thus, this study was aimed to study whether different fibroid locations influence clinical outcome following UAE and fibroids were grouped according to whether they affected the endometrium.

## Materials and methods

### Study design and participants

A retrospective cohort study was conducted on patients who underwent UAE for symptomatic uterine fibroid at the Radiology Department of our hospital between December 2016 and January 2023. Patients with available pre-treatment and 6-month post-treatment data, including MRI images, FIGO classification, uterine volume, and uterine fibroid volume, were included for analysis. This study received approval from the Ethics Committee of our hospital prior to chart review, and written informed consent was waived due to the retrospective study design (approval number: E23-0396).

### Imaging protocol and FIGO classification

All patients underwent pelvic MRI (T2-weighted) for visualization of uterine fibroid and arteries before UAE at the Radiology Department of our hospital, following an established protocol. Detailed protocol information has been described elsewhere [17, 18]. The margin of pretreatment volume of uterine and fibroid was delineated by experienced radiologists (assistant professor with 14 years of experience and professor with 39 years of experience). The volume was calculated for each patient using the volume analyzer of the Synapse Vincent system based on imported sagittal T2-weighted images (FUJIFILM Medical Co., Ltd., Tokyo, Japan). Images were used to assess the submucosal, intramural, and subserous components of uterine fibroid, which were then used to calculate protrusion rates. Submucosal uterine fibroids include those with pedunculated intracavity extension (FIGO type 0), a submucosal protrusion rate of ≥ 50% (FIGO type 1), and a submucosal protrusion rate of < 50% (FIGO type 2). Uterine fibroids with 100% intramural components include those in contact with, but not extending into, the endometrium (FIGO type 3) and those not in contact with the endometrial and subserous layers (FIGO type 4). Subserous uterine fibroids include those with an intramural protrusion rate of ≥ 50% (FIGO type 5), an intramural protrusion rate of < 50% (FIGO type 6), and pedunculated subserous component (FIGO type 7). Patients whose uterine fibroid was other than FIGO type 0–7, i.e. hybrid were excluded from the analysis, and for patients with multiple uterine fibroids, the volume of the largest fibroid was evaluated for this study. Note, in our hospital, patients with FIGO 0 and 1 received hysteroscopic myomectomy, therefor no data was available for FIGO 0 and 1 treated by UAE.

### UAE protocol

UAE protocol followed the same procedure as previously described [18]. In brief, UAE was performed bilaterally on patients under conscious sedation with local anesthesia by radiologist with 13 years of experience as of December 2016. Briefly, a PIG-S4 catheter (Medikit Angiography Catheter MH; Medikit Co., Ltd., Tokyo, Japan) guided by an angled wire was placed in the abdominal aorta for initial aortography. Subsequently, a 4-Fr MOHRI-1 catheter (Medikit Angiography Catheter; Medikit Co., Ltd. Tokyo, Japan) followed by a microcatheter (2.6-Fr Masters HF; ASAHI Intecc, Aichi, Japan) over a micro-guidewire (0.016 inch; Piolax Medical Devices, Kanagawa, Japan) was used to reach the beginning and the proximal ascending segment of the uterine artery, respectively. After confirming the target artery’s perfusion area using iopromide 300 (Ultravist®; Bayer AG, Leverkusen, Germany), embolic material (Embosphere®; Merit Medical, UT, USA) (2 mL) suspended in a mixture of 9 mL of sterile saline and 9 mL of contrast agent was administered to the site (the final embolic material dilution in solution was 1:20). Typically, the embolization starts with 500–700 µm particles for small arteries, and if necessary, larger particles (700–900 µm) were used to achieve the desired occlusion till the end of the procedure. An aortogram was performed to confirm the success of the UAE. Pain control consisted of 1 mg of fentanyl and 2.5 mg of dropleton plus 20 ml of saline, administered at a rate of 4 ml per hour depending on pain. A separate route for sedation was used to administer up to 50 mg of Atarax P intravenously.

### Data analysis

In this study, patients were categorized into two groups based on the location of their largest fibroid, either FIGO 2/3, or FIGO 4 and above (4–7). The menstrual pain and flow were assessed on a scale of 0 (none) to 10 (worst ever experienced before treatment) at each post-procedural follow-up visit. The volume of the uterine and fibroid were assessed based on MR images, following the same procedure as previously described [18]. For patients with multiple fibroids, in addition to measuring the largest myoma nodule, we also measured the volume of the entire uterus to determine the effectiveness of the UAE. Changes in uterine and fibroid volume at 6 months after UAE were expressed as a percentage of pre-treatment volume, while changes in menstrual flow and pain 6 months after treatment were expressed in points. Treatment satisfaction representing HR-QoL was assessed on a 5-point Likert scale (ranging from very unsatisfied to very satisfied) at pre-treatment outpatients visit and at post-treatment visit. The between-group differences were tested using the Welch two sample t-test, one-way analysis of variance, Fisher’s exact test, and Chi’s square test, as indicated. Differences with a *p*-value below 0.05 were considered statistically significant. All statistical analyses were performed using R 4.2.2 (R Core Team, 2022).

## Results

### Participant characteristics

A total of 149 premenopausal patients who received UAE for uterine fibroids classified as FIGO types 2–7 were included for analysis, with a mean age of 45.7 ± 2.7 years (Table 1). The overall mean and range of pre-UAE fibroid and uterine volumes was (mean ± SD [min–max]): 389.7 ± 299.8 [0.6–1,561.9]cm^3^ and 909.1 ± 438.9 [191.1–2,630.2] cm^3^, respectively. The mean baseline menstrual flow and pain index were 8.6 ± 2.1 [0–10] and 5.0 ± 3.5 [0–10], respectively. There were 57 fibroids classified as FIGO 2/3 (38.3%) and 92 as FIGO 4–7 (61.7%). Note, there were no FIGO 0 or 1 because uterine fibroids classified as FIGO 0 and 1 would receive hysteroscopic myomectomy in our hospital. The mean age of the FIGO 2/3 group was slightly higher than the FIGO 4–7 group (46.3 ± 2.2 *vs* 45.4 ± 3.0 years, *p* = 0.050). At baseline, the mean menstrual flow index differed significantly between fibroid locations (9.4 ± 1.4 *vs* 8.0 ± 2.3 points, *p* < 0.001). Patients with FIGO 2 had a higher menstrual flow index at pre-treatment (Supplemental Table 1). However, no difference was observed in (mean ± SD [min–max]) baseline menstrual pain index (FIGO 2/3: 5.5 ± 3.2 [0–10] *vs* FIGO 4–7: 4.7 ± 3.6 [0–10], *p* = 0.181), fibroid volume (FIGO 2/3: 433.3 ± 353.5 [51.9–1,561.9]cm^3^ *vs* FIGO 4–7: 362.6 ± 259.4 [0.6–1,175.0]cm^3^, *p* = 0.194), and uterine volume (FIGO 2/3: 861.7 ± 480.9 [191.1– 2,211.2]cm^3^ *vs* FIGO 4–7: 938.4 ± 410.8 [201.8–2,630.2]cm^3^, *p* = 0.320).

**Table 1:** Characteristics of patients who underwent UAE, by FIGO classification

**Variable**		**FIGO classification**^**a**^		***p*-value**^†^
	**Overall** *N* = 149	**FIGO 2/3** *N* = 57	**FIGO 4–7** *N* = 92	
**Age at UAE, mean ± SD [min – max]**	45.7 ± 2.7 [36.0—53.0]	46.3 ± 2.2 [42.0—52.0]	45.4 ± 3.0 [36.0—53.0]	**0.050**
**FIGO of the largest fibroid, n (%)**				** < 0.001**
2	11 (7%)	11 (19%)	0 (0%)	
3	46 (31%)	46 (81%)	0 (0%)	
4	22 (15%)	0 (0%)	22 (24%)	
5	27 (18%)	0 (0%)	27 (29%)	
6	26 (17%)	0 (0%)	26 (28%)	
7	17 (11%)	0 (0%)	17 (18%)	
**Pre-UAE Symptom Status**				
Menstrual flow index, mean ± SD [min – max]	8.6 ± 2.1 [0—10]	9.4 ± 1.4 [4–10]	8.0 ± 2.3 [0—10]	** < 0.001**
Menstrual pain index, mean ± SD [min – max]	5.0 ± 3.5 [0—10]	5.5 ± 3.2 [0—10]	4.7 ± 3.6 [0—10]	0.181
Fibroid volume (cm^3^), mean ± SD [min – max]	389.7 ± 299.8 [0.6—1,561.9]	433.3 ± 353.5 [51.9—1,561.9]	362.6 ± 259.4 [0.6—1,175.0]	0.194
Uterine volume (cm^3^), mean ± SD [min – max]	909.1 ± 438.9 [191.1—2,630.2]	861.7 ± 480.9 [191.1—2,211.2]	938.4 ± 410.8 [201.8—2,630.2]	0.320

*FIGO* the International Federation of Gynecology and Obstetrics, *SD* standard deviation, *UAE* uterine artery embolization

^†^Welch Two Sample t-test; Fisher’s exact test

^a^Patients were categorized into two groups based on the FIGO classification of their largest fibroid: Those with a classification of either 2 or 3, and those classified between 4 and 7. There were no patients with a FIGO classification of 0 or 1

### Post-treatment improvements and treatment satisfaction

Six months after UAE, the overall change in menstrual flow and pain index was -3.0 ± 2.4 and -2.1 ± 2.6 points, respectively (Table 2). Furthermore, the treated fibroid shrank by 45.5 ± 19.8%, and the size of the uterus was reduced by 33.8 ± 14.0%. Very few patients were very dissatisfied (2%) or somewhat dissatisfied (2%) with the treatment outcome, while those who were satisfied or very satisfied represented 88% of the total study cohort. Patients in the FIGO 2/3 group achieved a significantly improvement in menstrual flow index (-3.7 ± 2.6 *vs*-2.6 ± 2.2 points, *p* = 0.035), fibroid volume (-54.7 ± 21.7 *vs* -39.8 ± 16.2%, *p* < 0.001), and uterine volume (-38.2 ± 16.3 *vs* -31.1 ± 11.6%, *p* = 0.008) compared to the FIGO 4–7 group. Furthermore, the level of improvement was significantly higher for FIGO 2 compared with FIGO 3 (Supplemental Table 2). The patients in the FIGO 2/3 group improved by -2.6 ± 2.7 points in menstrual pain index, and the patients in the FIGO 4–7 group also achieved a comparable reduction by -1.8 ± 2.5 points (*p* = 0.099).

**Table 2:** Changes after UAE, by FIGO classification

**Variable**		**FIGO classification**^**b**^		***p*-value**^†^
	**Overall** *N* = 149	**FIGO 2/3** *N* = 57	**FIGO 4–7** *N* = 92	
**Changes in symptoms after UAE**				
Menstrual flow index, mean ± SD [min – max]	-3.0 ± 2.4 [-8—3]	-3.7 ± 2.6 [-8—3]	-2.6 ± 2.2 [-7—2]	**0.035**
Menstrual pain index, mean ± SD [min – max]	-2.1 ± 2.6 [-10—3]	-2.6 ± 2.7 [-8—3]	-1.8 ± 2.5 [-10—2]	0.099
Fibroid volume (%), mean ± SD [min – max]	-45.5 ± 19.8 [-97.4—25.9]	-54.7 ± 21.7 [-97.4—-12.2]	-39.8 ± 16.2 [-76.7—25.9]	** < 0.001**
Uterine volume (%), mean ± SD [min – max]	-33.8 ± 14.0 [-75.5—10.5]	-38.2 ± 16.3 [-75.5—-4.2]	-31.1 ± 11.6 [-59.1—10.5]	**0.008**
**Treatment satisfaction level, n**^**a**^** (%)**				0.129
Very satisfied	43 (41%)	17 (44%)	26 (39%)	
Satisfied	50 (47%)	19 (49%)	31 (46%)	
Neutral	9 (8%)	1 (3%)	8 (12%)	
Somewhat dissatisfied	2 (2%)	2 (5%)	0 (0%)	
Very dissatisfied	2 (2%)	0 (0%)	2 (3%)	

*FIGO* the International Federation of Gynecology and Obstetrics, *SD* standard deviation, *UAE* uterine artery embolization

^†^Welch two sample t-test; Fisher’s exact test

^a^Satisfaction was measured on a five-point scale. Out of all the subjects analyzed, responses were not obtained from 43 individuals, leading to a total of 106 respondents

^b^Patients were categorized into two groups based on the FIGO classification of their largest fibroid: Those with a classification of either 2 or 3, and those classified between 4 and 7. There were no patients with a FIGO classification of 0 or 1

**Fig. 1:**
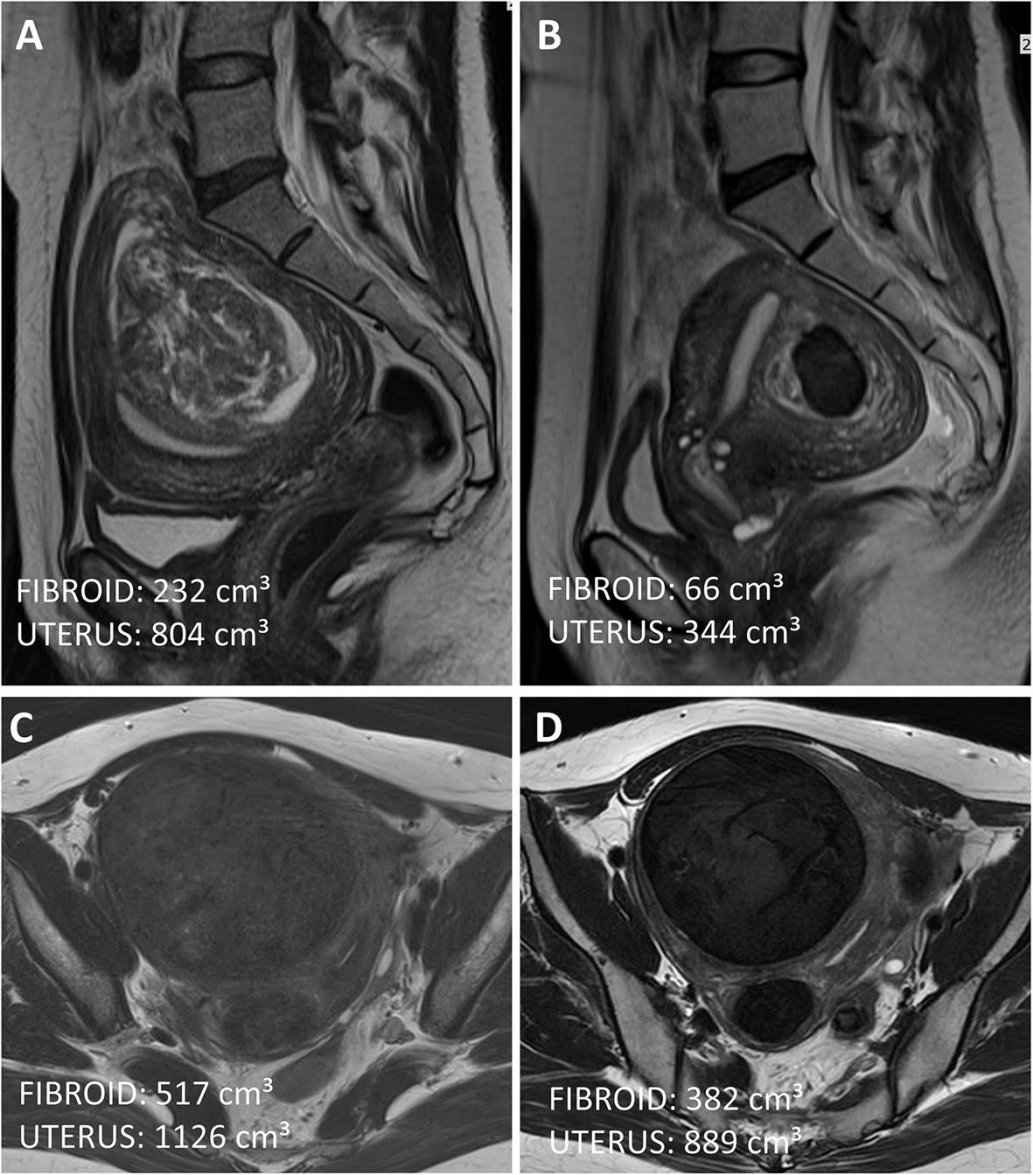
MRI assessment of the uterine and fibroid volume post-UAE. FIGO 3 fibroid showed a 71% volume, and uterine volume decreased by 57% from baseline (**A**) to 6 months after UAE (**B**). FIGO 4 fibroid had a 26% volume reduction, with a 21% decrease in uterine volume from baseline (**C**) to 6 months after UAE (**D**). Menstrual symptoms improved notably after UAE; the patient with FIGO 3 fibroid experienced a reduction in menstrual flow (from 6/10 to 3/10) and pain (from 2/10 to 0/10). The patient with FIGO 4 fibroid reported a decrease in menstrual flow (from 10/10 to 7/10) and pain (from 10/10 to 5/10) at the 6-month follow-up

A total of 106 patients completed the treatment satisfaction survey at follow-up visits post-treatment. Both groups were comparably satisfied with the treatment outcome (*p* = 0.129), with 93% of patients in the FIGO 2/3 group and 85% of the patients in FIGO 4–7 group being satisfied or very satisfied with the treatment results. MRI image of two cases illustrating the reduction in uterine fibroid volume 6 months after UAE is shown in Fig. 1.

### Subgroup analysis

We further compared the treatment outcomes in relation to each FIGO staging. The mean age did not differ significantly across the 6 patient groups (Table 3). The mean menstrual flow index reduction ranged from 5.8 (FIGO 2) to 2.1 (FIGO5), and no statistically significant intergroup difference was noted (*p* = 0.205). Similarly, the mean reduction in menstrual pain index did not differ across the FIGO groups (range: 2.8 [FIGO 2] to 1.1 [FIGO6]). The fibroid and uterine volume improvements, however, differed significantly, with the FIGO 2 group achieving the largest reduction in both fibroid volume (61.6 ± 32.1%) and uterine volume (38.5 ± 24.0%), and the FIGO 6 group having the smallest reduction in both fibroid (33.9 ± 15.2%) and uterine (26.7 ± 10.3%) volume. The majority of patients in all FIGO groups were either satisfied (range: 33% [FIGO 2] to 73% [FIGO 7]) or very satisfied (range: 17% [FIGO 7] to 47% [FIGO 6]), and the distribution of treatment satisfaction did not differ significantly across groups (*p* = 0.088).

**Table 3:** Changes after UAE, by individual FIGO classification

**Variable**	**FIGO 2** *N* = 11	**FIGO 3** *N* = 46	**FIGO 4** *N* = 22	**FIGO 5** *N* = 27	**FIGO 6** *N* = 26	**FIGO 7** *N* = 17	***p*-value**^†^
**Age at UAE, mean ± SD [min – max]**	46.8 ± 2.3 [43.0—49.0]	46.1 ± 2.2 [42.0—52.0]	46.0 ± 2.3 [42.0—49.0]	45.3 ± 4.0 [36.0—53.0]	44.7 ± 2.5 [41.0—50.0]	45.8 ± 2.2 [41.0—49.0]	0.205
**Changes in symptoms after UAE**							
Menstrual flow index, mean ± SD [min – max]	-5.8 ± 1.5 [-8—-5]	-3.5 ± 2.7 [-8—3]	-2.9 ± 2.4 [-7—1]	-2.1 ± 2.5 [-7—2]	-3.0 ± 2.0 [-6—0]	-2.6 ± 1.9 [-7—0]	0.074
Menstrual pain index, mean ± SD [min – max]	-2.8 ± 3.6 [-8—0]	-2.6 ± 2.6 [-8—3]	-2.5 ± 3.1 [-8—2]	-1.7 ± 2.7 [-10—1]	-1.1 ± 1.6 [-5—0]	-1.8 ± 2.2 [-7—0]	0.321
Fibroid volume (%), mean ± SD [min – max]	-61.6 ± 32.1 [-97.4—-12.2]	-53.6 ± 19.9 [-92.5—-21.9]	-39.4 ± 19.1 [-65.7—25.9]	-41.3 ± 13.7 [-70.6—-17.6]	-33.9 ± 15.2 [-67.5—-9.6]	-47.5 ± 15.1 [-76.7—-23.7]	** < 0.001**
Uterine volume (%), mean ± SD [min – max]	-38.5 ± 24.0 [-75.5—-4.2]	-38.1 ± 15.1 [-75.1—-12.3]	-32.5 ± 10.0 [-47.7—-13.3]	-30.5 ± 12.1 [-46.1—10.5]	-26.7 ± 10.3 [-53.3—-12.8]	-37.0 ± 13.0 [-59.1—-17.3]	**0.017**
**Treatment satisfaction level, n**^**a**^** (%)**							0.088
Very satisfied	1 (33%)	16 (44%)	8 (40%)	7 (41%)	9 (47%)	2 (18%)	
Satisfied	1 (33%)	18 (50%)	8 (40%)	8 (47%)	7 (37%)	8 (73%)	
Neutral	0 (0%)	1 (3%)	3 (15%)	1 (6%)	3 (16%)	1 (9%)	
Somewhat dissatisfied	1 (33%)	1 (3%)	0 (0%)	0 (0%)	0 (0%)	0 (0%)	
Very dissatisfied	0 (0%)	0 (0%)	1 (5%)	1 (6%)	0 (0%)	0 (0%)	

*FIGO* the International Federation of Gynecology and Obstetrics, *SD* standard deviation, *UAE* uterine artery embolization

^†^One-way ANOVA; Pearson’s Chi-squared test

^a^Satisfaction was measured on a five-point scale. Out of all the subjects analyzed, responses were not obtained from 43 individuals, leading to a total of 106 respondents

## Discussion

The present study reveals that uterine fibroids classified as FIGO 2/3 exhibited significantly greater reductions compared to those classified as FIGO 4–7 at 6 months post-UAE. Specifically, the highest reduction in fibroid and uterine volume was observed in patients with FIGO 2 fibroids, and the lowest was seen in patients with FIGO 6 fibroids. This suggests that fibroids affecting the endometrial components (FIGO 2) or in contact with the endometrium (FIGO 3) respond better to UAE compared to fibroids without endometrial contact (FIGO 4–7).

Several studies have reported significant fibroid volume reduction and symptom improvement 3 months postintervention in women undergoing UAE for symptomatic uterine fibroids [19, 20], without affecting healthy uterine tissue [21]. A meta-analysis indicated that UAE resulted in a size reduction of 53.2% in uterine fibroids < 10 cm, with slightly less reduction (48%) observed in larger fibroids [16], and few studies reported the lack of a causal relationship between baseline estrogen, luteinizing hormone, or follicle-stimulating hormone with UAE results in uterine fibroids at different FIGO stages [22, 23]. Nevertheless, very few studies have explored the influence of uterine fibroid location based on FIGO classification and post-UAE clinical outcomes, safety, or treatment satisfaction.

The finding that treatment efficacy differed by FIGO stage is consistent with a recent study by Ito et al. [23], which analyzed the treatment response of 42 symptomatic women (mean age 45.2 years) who underwent UAE for uterine fibroids with and without submucosal involvement. In their study, the volume reduction rate at 3 and 6 months was found to be significantly higher in women with fibroids within or in contact with the uterine submucosa (FIGO 0–2 and FIGO 3) compared to those outside the submucosa (FIGO 4–7). Symptom improvement was observed in almost all patients, with 26 of the 29 patients showing decreased menstrual flow [23]. Similarly, a study that evaluated the risk factors for post-UAE fibroid expulsion (implying fibroid volume loss) found that patients with FIGO 0–2 fibroids were more likely to experience fibroid expulsion, with or without symptoms, compared to those with FIGO 3–7 fibroids (*p* < 0.01) [24]. In addition, they reported that FIGO stage was not associated with the rate of fibroid expulsion requiring procedures or surgery [24]. In contrast, a study by Cappelli et al. [25], which evaluated the 1-year outcome of middle-aged women who received UAE for symptomatic fibroids with FIGO stages ranging from 0 to 6, reported that despite an overall 42.6% reduction in dominant fibroid diameter, neither FIGO stage nor fibroid diameter was significantly associated with diameter reduction. Furthermore, reduced symptom severity was self-reported by only 19% of the patients at 1-year post-procedure [25]. However, none of these studies reported patient-perceived treatment satisfaction, and few examined improvements in quality of life in relation to FIGO staging. Our study demonstrated that FIGO 2/3 uterine fibroids exhibited 15% more volume reduction and 7% more symptom improvement compared to FIGO 4–7 at 6 months after UAE. Despite the difference in volume reduction levels, patients expressed satisfaction with the treatment, regardless of their FIGO stage.

Investigations into predictive markers for post-procedural clinical and life quality outcomes after UAE have yielded varied results. Older age, concomitant adenomyosis, and high intramural involvement were identified as significant predictors for poor outcomes, defined as the occurrence of persistent or recurrent symptoms, or requiring additional intervention in a recent study [26]. This is in contrast with findings from an earlier report, where lesion features, including fibroid location and size, rather than patient characteristics, were associated with UAE-induced leiomyoma infarction results [27]. A study evaluating the predictive values of MRI features of uterine leiomyoma indicated that a submucosal location, a baseline leiomyoma volume of < 58 cm^3^, and hyperintensity on T2-weighted MRI or subtraction imaging were predictors of a greater UAE-associated leiomyoma volumetric response [28]. Microvascular imaging that provides more detailed microvessel information of targeted uterine fibroids on Doppler ultrasound was shown to be a good predictor for > 30% fibroid volume reduction [29]. The use of baseline MRI-derived apparent diffusion coefficients was also proposed to be correlated with fibroid volume reduction at 6 months after UAE [30]. Recent research efforts have utilized machine-learning models trained using MRI features from routine T1-weighted contrast-enhanced (T1C) and T2-weighted sequences to achieved high accuracy in predicting successful symptom improvement [31]. So far, there is currently a lack of consensus on risk factors or predictors for outcomes after UAE, and various treatment outcome definitions have been used despite seemingly comparable baseline patient selection criteria.

The present study is one of the few studies that combined both imaging and clinical data, however, it is limited by its single-centered study design with limited number of patients in each category of FIGO fibroids. Furthermore, even though the HR-QoL were collected prospective at pre-treatment and at post-treatment follow-up outpatient visits, the survey was not validated. Additionally, due to the retrospective nature of the study, data on pregnancy history, smoking status, and comorbidities at the time of UAE were not available for all patients, and thus we were not able to further explore the potential causes of patient dissatisfaction among those who reported dissatisfied with post-UAE treatment outcomes. Therefore, the results remain to be validated with prospective multi-center studies with a large sample size and longer follow-up, using validated outcome measures such as that of Uterine Fibroid Symptom Health-Related Quality of Life Questionnaire.

## Conclusions

UAE is effective in reducing uterine fibroid, and the majority of patients expressed satisfaction with the treatment outcomes regardless of FIGO staging. Our findings demonstrated that the postoperative lesion volume reductions and symptom improvements following UAE for fibroid were particularly better for FIGO 2/3 compared to FIGO 4–7, which suggests that UAE may have better outcomes in fibroid affecting the endometrium. A multicenter study with larger sample size is warranted.

## Supplementary Information

Supplementary Material 1.

## Data Availability

The data that support the findings of this study are available on request from the corresponding author.

## Abbreviations

FIGO: International Federation of Gynecology and Obstetrics
HR-QoL: Health-related quality of life
MRI: Magnetic resonance imaging
T1C: T1-weighted contrast-enhanced
UAE: Uterine artery embolization
