# Supplementary material for: Postoperative symptom changes following uterine artery embolization for uterine fibroid based on FIGO classification
Source: CVIR Endovasc. 2025 Jan 29;8:9. doi: 10.1186/s42155-025-00520-7 (PMC11780029; doi:10.1186/s42155-025-00520-7)
Supplement: Supplementary file 1 — Supplementary Material 1. [file 42155_2025_520_MOESM1_ESM.docx]

Supplemental Table 1. Characteristics of patients who underwent UAE, by FIGO classification (2, 3, 4 and above)

| **Variable** |  | **FIGO classification*** | | | **p-value**^†^ |
| --- | --- | --- | --- | --- | --- |
|  | **Overall**  N = 149 | **FIGO 2**  N = 11 | **FIGO 3**  N = 46 | **FIGO 4-7**  N = 92 |  |
| **Age at UAE, mean ±** **SD**  **[min – max]** | 45.7 ± 2.7  [36.0 - 53.0] | 46.8 ± 2.3  [43.0 - 49.0] | 46.1 ± 2.2  [42.0 - 52.0] | 45.4 ± 3.0  [36.0 - 53.0] | 0.138 |
| **FIGO of the largest fibroid, n (%)** |  |  |  |  | **<0.001** |
| 2 | 11 (7%) | 11 (100%) | 0 (0%) | 0 (0%) |  |
| 3 | 46 (31%) | 0 (0%) | 46 (100%) | 0 (0%) |  |
| 4 | 22 (15%) | 0 (0%) | 0 (0%) | 22 (24%) |  |
| 5 | 27 (18%) | 0 (0%) | 0 (0%) | 27 (29%) |  |
| 6 | 26 (17%) | 0 (0%) | 0 (0%) | 26 (28%) |  |
| 7 | 17 (11%) | 0 (0%) | 0 (0%) | 17 (18%) |  |
| **Pre-UAE Symptom Status** |  |  |  |  |  |
| Menstrual flow index, mean **±** SD  [min – max] | 8.6 ± 2.1  [0 - 10] | 10.0 ± 0.0  [10 - 10] | 9.4 ± 1.4  [4 - 10] | 8.0 ± 2.3  [0 - 10] | **0.002** |
| Menstrual pain index, mean **±** SD  [min – max] | 5.0 ± 3.5  [0 - 10] | 6.3 ± 4.1  [1 - 10] | 5.5 ± 3.2  [0 - 10] | 4.7 ± 3.6  [0 - 10] | 0.392 |
| Fibroid volume (cm^3^), mean **±** SD  [min – max] | 389.7 ± 299.8 [0.6 - 1,561.9] | 387.5 ± 510.1 [51.9 - 1,440.2] | 444.3 ± 311.5 [67.0 - 1,561.9] | 362.6 ± 259.4  [0.6 - 1,175.0] | 0.323 |
| Uterine volume (cm^3^), mean **±** SD  [min – max] | 909.1 ± 438.9 [191.1 - 2,630.2] | 754.0 ± 616.0 [191.1 - 1,874.6] | 887.5 ± 447.2 [254.5 - 2,211.2] | 938.4 ± 410.8 [201.8 - 2,630.2] | 0.390 |

*Patients were categorized into three groups based on the FIGO classification of their largest fibroid: Those with a classification of 2, those with a classification of 3, and those classified between 4 and 7. There were no patients with a FIGO classification of 0 or 1. ^†^Welch Two Sample t-test; Fisher's exact test.

FIGO, the International Federation of Gynecology and Obstetrics; SD, standard deviation; UAE, uterine artery embolization.

| **Variable** |  | **FIGO classification*** | | | **p-value**^†^ |
| --- | --- | --- | --- | --- | --- |
|  | **Overall**  N = 149 | **FIGO 2**  N = 11 | **FIGO 3**  N = 46 | **FIGO 4-7**  N = 92 |  |
| **Changes in symptoms after UAE** |  |  |  |  |  |
| Menstrual flow index, mean **±** SD  [min – max] | -3.0 ± 2.4  [-8 - 3] | -5.8 ± 1.5  [-8 - -5] | -3.5 ± 2.7  [-8 - 3] | -2.6 ± 2.2  [-7 - 2] | **0.015** |
| Menstrual pain index, mean **±** SD  [min – max] | -2.1 ± 2.6  [-10 - 3] | -2.8 ± 3.6  [-8 - 0] | -2.6 ± 2.6  [-8 - 3] | -1.8 ± 2.5  [-10 - 2] | 0.244 |
| Fibroid volume (%), mean **±** SD  [min – max] | -45.5 ± 19.8  [-97.4 - 25.9] | -61.6 ± 32.1  [-97.4 - -12.2] | -53.6 ± 19.9 [-92.5 - -21.9] | -39.8 ± 16.2  [-76.7 - 25.9] | **<0.001** |
| Uterine volume (%), mean **±** SD  [min – max] | -33.8 ± 14.0  [-75.5 - 10.5] | -38.5 ± 24.0  [-75.5 - -4.2] | -38.1 ± 15.1 [-75.1 - -12.3] | -31.1 ± 11.6  [-59.1 - 10.5] | **0.015** |
| **Treatment satisfaction level, n^a^ (%)** |  |  |  |  | 0.125 |
| Very satisfied | 43 (41%) | 1 (33%) | 16 (44%) | 26 (39%) |  |
| Satisfied | 50 (47%) | 1 (33%) | 18 (50%) | 31 (46%) |  |
| Neutral | 9 (8%) | 0 (0%) | 1 (3%) | 8 (12%) |  |
| Somewhat dissatisfied | 2 (2%) | 1 (33%) | 1 (3%) | 0 (0%) |  |
| Very dissatisfied | 2 (2%) | 0 (0%) | 0 (0%) | 2 (3%) |  |

Supplemental Table 2. Changes after UAE, by FIGO classification (2, 3, 4 and above)

*Patients were categorized into two groups based on the FIGO classification of their largest fibroid: Those with a classification of 2, those with a classification of 3, and those classified between 4 and 7. There were no patients with a FIGO classification of 0 or 1.

^†^One-way ANOVA; Fisher's exact test.

**^a^** Satisfaction was measured on a five-point scale. Out of all the subjects analyzed, responses were not obtained from 43 individuals, leading to a total of 106 respondents

FIGO, the International Federation of Gynecology and Obstetrics; SD, standard deviation; UAE, uterine artery embolization.
